# New insights into the pathophysiology of the small airways in asthma

**DOI:** 10.4103/1817-1737.30361

**Published:** 2007

**Authors:** Qutayba Hamid, Meri K. Tulic

**Affiliations:** Meakins-Christie Laboratories, McGill University, Montreal, QC Canada; * *Division of Cell Biology, Telethon Institute for Child Health Research and Centre for Child Health Research, University of Western Australia, Perth, Western Australia*

**Keywords:** Allergic inflammation, asthma, distal airways, parenchyma

## Abstract

Asthma is a lung disease characterized by inflammation and remodeling of the airways, which leads to airflow obstruction and symptoms of wheeze, chest tightness, cough and dyspnea. It is now widely accepted that airway inflammation and remodeling occur not only in the central airways but also in the small airways and even in the lung parenchyma. Inflammation of the distal lung can be observed even in mild asthmatics with normal or noncompromised lung function. Moreover, the small airways and the lung parenchyma can produce many Th2 cytokines and chemokines involved in initiation and perpetuation of the inflammatory process. In addition, the distal parts of the lung have been recognized as a predominant site of airflow obstruction in asthmatics. In fact, the inflammation at this distal site has been described as more severe when compared to the large airway inflammation, and evidence of remodeling in the lung periphery is emerging. Recognition of asthma as a disease of the entire respiratory tract has an important clinical significance, highlighting the need to also consider the distal lung as a target in any therapeutic strategy for effective treatment of this disease.

Asthma is a chronic inflammatory disease of the lung characterized by episodic airway obstruction and increased bronchial responsiveness. The major pathological and structural features of asthma include epithelial shedding, airway smooth muscle hypertrophy and hyperplasia, mucous gland hyperplasia, sub-epithelial fibrosis and infiltration of the bronchial wall with inflammatory cells.[1] The concept that inflammation is a major component of asthmatic pathology was established more than 100 years ago, and these early studies have used autopsy specimens to study the macroscopic morphological and histological changes within the large asthmatic airways.[2] That the distal airways and the lung parenchyma play a role in asthma has been suggested by experiments in which the physiological behavior of the lung has been investigated.[3,4] These early studies, originally conducted at the Meakins-Christie laboratories, focused attention on the role of the distal airways in asthma; however, investigation in this area lagged because of the difficulties in examining these peripheral structures directly. Since then, the development of new techniques of physiological measurements have focused on assessing the role of lung periphery, and this distal site has now been recognized as a predominant site of airflow obstruction in asthmatics.[5–8] Introduction of fiber-optic bronchoscopy technique has enabled us to obtain small human endobronchial biopsies from the large airways of asthmatic patients; and together with the recent applications of molecular pathology techniques including immunocytochemistry and *in situ* hybridization to endobronchial biopsies and lavage fluid, these technological changes have had a profound impact on our understanding of the pathogenesis of bronchial asthma.[9,10] Recent studies utilizing these approaches in surgically resected lung tissue,[11–13] postmortem lung specimens[14–16] and transbronchial biopsies[17,18] have demonstrated that similar but more severe inflammatory and structural changes are also occurring in the distal lung[15,19,20] and in the lung parenchyma of asthmatic patients.[17]

It is now accepted that in asthmatics, recruitment of inflammatory cells, in particular eosinophils and T cells, also occurs in the distal lung[11,12] and the lung parenchyma.[17] In addition, in asthmatics there is an abundance of Th2-type cytokines and chemokines present at this distal site.[12,13] These pathological findings may prove to be extremely important as the total volume and combined surface area of the distal airways are much greater than the combined volume and the surface area of the large airways.[21] This clearly suggests that any changes developing in the distal lung and the parenchyma in patients with asthma will have a dramatic effect on the pathogenesis and treatment of this disease.

The current therapeutic challenge is to develop better inhalation technologies to improve the delivery of anti-inflammatory agents to the lung periphery. In the past, this may have been the neglected site of treatment in asthmatics. This review article aims to evaluate the pathological and physiological evidence presented in the literature to date, which outlines the contribution of the distal lung and the lung parenchyma to the pathophysiology of asthma. We will also explore some of the new technologies of drug delivery and the efficacy and safety of these new formulations for the asthmatic patients. We will also address the important questions which remain to be answered regarding the distal airways and the future directions in treatment of this disease.

## Inflammation and Remodeling in the Small Airways

Asthma is characterized by infiltration of central airways with inflammatory cells and upregulation of Th2-type cytokines, in particular IL-5, IL-4, IL-9 and IL-13. Largely these observations stem from studies that sample central airways, as bronchoscopic sampling was largely limited to proximal airway sites only. For this reason, the pathologic process that occurs in the distal airways has been less well defined. The early studies surrounding the role of the peripheral or the distal airways in pathophysiology of asthma originated from autopsy studies.[14,15,22] These studies demonstrated that the entire length of the airway is involved in asthma and not only the central airways as originally proposed.[7,23–25] Carroll and colleagues have carefully examined the distribution of inflammatory cells throughout the bronchial tree of both fatal and nonfatal cases of asthma and have shown increased numbers of lymphocytes and eosinophils uniformly distributed throughout the large and distal airways of mild and severe asthmatics when compared to control cases.[14] Similar infiltration of T cells, macrophages and eosinophils into the proximal and distal lung tissues has also been reported in rare cases of sudden asthma death (dying within 1 h after the onset of their symptoms)[15] and in soybean dust-induced asthma.[20]

Using resected lung specimens from asthmatic and non-asthmatic patients who underwent thoracic surgery, we have similarly demonstrated that the inflammatory response in asthma is not restricted to the proximal airways but is also seen in the distal lung.[11] We reported increased number of CD3+ T cells and major basic protein (MBP) + eosinophils in not only the large airways (>2 mm internal diameter) but also in the distal airways (<2 mm internal diameter) of asthmatic patients when compared to healthy controls. In fact, greater number of activated eosinophils was seen in the airways <2 mm internal diameter than in the airways >2 mm internal diameter, suggesting that similar but more severe inflammatory process is present in the distal airways.[26] In the same cohort of patients, we have also shown increased IL-5 and IL-4 mRNA-positive cells in the distal airways of asthmatic subjects compared to non-asthmatic controls,[12] and more importantly, the expression of IL-5 mRNA was increased in the distal airways compared to the large airways.[12] Numbers of cells expressing IL-2 and IFN-γ mRNA remained unchanged in these subjects when compared to controls. Furthermore, simultaneous *in situ* hybridization and immunocytochemistry has demonstrated 85% of the IL-5 mRNA-positive cells in the distal airways to be CD3+ T cells, similar to the proportion found in the large airways (81%).[12] In addition, we have recently shown increased eotaxin and monocyte chemotactic protein -4 mRNA expression in the distal airway epithelium of asthmatics compared to non-asthmatics and the number of chemokine positive cells in the distal airways of these patients to correlate with the number of MBP + eosinophils at this same site.[13]

The use of lung sections has allowed examination of the smooth muscle cells. Haley and colleagues have evaluated the distribution of CD45+ cells within the subepithelial regions of the airway wall and have shown that the distal asthmatic airways had the majority of their CD45+ leukocytes and eosinophils in the ‘outer’ airway wall regions (‘outer’ defined as the area between the smooth muscle and the alveolar attachments), whereas the greatest density of eosinophils in the large asthmatic airways was in the ‘inner’ airway wall regions (‘inner’ area defined as the area between the basement membrane and the smooth muscle).[25] In addition, they have demonstrated that the distal asthmatic airways showed a greater number of CD45+ cells and eosinophils in their ‘outer’ compared with their ‘inner’ airway wall regions.[25] These differences highlight the regional variations in inflammatory cell distribution within the asthmatic airway wall, and these differences appear to be disease specific as such variations were not observed in patients with cystic fibrosis.[25] The different regional organization of inflammatory cells throughout the tracheo-bronchial tree may be attributed to the differences in mechanisms of inflammatory cell recruitment and/or differences in chemokine and cytokine productions between these regions. These differences may influence the physiological response caused by local production of pro-inflammatory mediators. In support of this claim, increased expression of eotaxin, a potent eosinophil chemoattractant, has been documented in the airway epithelial layer of the peripheral airways of asthmatics.[13] The smooth muscle itself has been proposed as a site of chemokine production in the distal airways.[25]

In patients who died of asthma, inflammation was found to extend well beyond the airway smooth muscle and is still significant around the pulmonary arterioles.[27] Kraft and colleagues have shown significant alveolar inflammation in patients with nocturnal asthma (NA), compared to patients with non-non-nocturnal asthma (NNA).[17] In those studies, both proximal airway endobronchial and distal alveolar tissue transbronchial biopsies were performed in the same patient at 4:00 p.m. and 4:00 a.m. Patients with nocturnal asthma (NA) had increased number of eosinophils *per* unit of lung volume in their lung parenchyma at 4:00 a.m. compared to patients without NA, and the NA patients had a greater number of eosinophils and macrophages in their alveolar tissue at 4:00 a.m. than at 4:00 p.m. Moreover, in NA patients, only alveolar but not central airway eosinophilia correlated with overnight reduction in lung function[17] and was associated with increased number of CD4+ cells in alveolar tissue of NA patients at 4:00 a.m. compared to NNA patients.[18,28] Although the number of CD4+ cells in the endobronchial lamina propria was higher than in the alveolar tissue, once again, only the alveolar CD4+ lymphocytes correlated with the predicted lung function at 4:00 a.m. (r = -0.68) and with the number of activated alveolar eosinophils (EG2+, r = 0.66).[18] In this same group of patients, NA was associated with reduced glucocorticoid receptor (GR)-binding affinity, reduced proliferation of peripheral blood mononuclear cells and decreased responsiveness to steroids when compared to NNA patients.[29] Those studies have demonstrated that the increased numbers of CD4+ cells in alveolar tissue in NA patients, as well as reduced GR-binding affinity and reduced steroid responsiveness, may be responsible for orchestrating eosinophil influx and exacerbations of symptoms in patients with NA. One of the proposed mechanisms which may be responsible for this phenomenon is upregulation in the expression of GR-β (Glucocorticoid receptor-β), which has previously been reported in steroid-insensitive subjects with severe asthma.[16] Christodoulopoulos and colleagues in our laboratory have characterized the expression of GR-β within the peripheral airways in cases of severe fatal asthma. They have demonstrated the main cells expressing GR-β (Glucocorticoid receptor-β) to be CD3+ T lymphocytes; and, to a lesser extent, eosinophils, neutrophils and macrophages.[16] Those results suggest that the increased number of GR-β (Glucocorticoid receptor-β) -positive cells in the distal airways of patients with fatal asthma may be associated with steroid resistance, contributing to asthma mortality.

Distal airway inflammation has been reported in severe symptomatic, steroid-dependent asthmatics.[30] Using endobronchial and transbronchial biopsies, Wenzel and colleagues reported persistent proximal and distal airway inflammation in these patients. Although the number of eosinophils was similar between severe asthmatics and normal controls, severe asthmatics had high numbers and percentages of neutrophils in their bronchoalveolar lavage and endobronchial and transbronchial biopsy specimens when compared with mild-to-moderate asthmatics, despite aggressive treatment with steroids.[30] In cases of fatal asthma, inflammatory cells make up a higher proportion of the exudate that occlude the smaller airways than the exudate from the larger airways.[31] From this evidence, it is thought that the inflammatory cell density in the distal airways of severe asthmatics may relate to their peripheral airway obstruction characteristic of this disease. The distal airway inflammation may cause an uncoupling of the parenchyma and airways due to the mechanical interdependence between these two compartments, leading to changes in overall lung mechanics in asthmatics.

Small airway mechanics: no longer the quiet zone of the lung Most of our knowledge of lung function in asthmatics comes from spirometric and plethysmographic measurements made during bronchoprovocation, and these measurements are dominated by large airway responsiveness. Since the volume and surface area of the lungs increase with increasing airway generations, the contribution of peripheral resistance to the total lung resistance was originally thought to be minimal.[21] Research conducted over three decades ago, using retrograde catheters in animals, demonstrated that the distal airways are pathways of small resistance, contributing to less than 10% of the total resistance to airflow in the lung models.[32,33] For this reason, the distal airways were originally described as the ‘quiet zone’ of the lungs in 1970.[34]

Since then, more sophisticated, frequency-dependent measurements of the peripheral airways have been developed. Invasive studies in mongrel dogs using alveolar capsules[35,36] or using the forced oscillation technique in rodents[37–40] have demonstrated that both airway and parenchymal compartments contribute to airway responsiveness. Methacholine (MCh)-induced increase in parenchymal resistance has also been reported in New Zealand white rabbits,[41,42] guinea-pigs[43] and rats.[38,44,45]

Invasive studies have been carried out in patients with asymptomatic asthma using a catheter-tipped micromanometer wedged into the lower lobe of the bronchus in order to partition central and peripheral airway resistance.[6] In those studies, the investigators constructed separate dose-response curves *in vivo* and showed a dose-dependent increase in both central and peripheral airway resistance in response to inhaled MCh in all patients studied. More recently, noninvasive methodologies for separating airway and parenchymal mechanics both in animals[46,47] and in humans[48–51] have been developed. Using the low-frequency forced oscillation technique, Hall and colleagues have demonstrated in infants that inhaled MCh alters both central and peripheral airway mechanics as well as lung parenchymal mechanics.[48] In normals and in patients with chronic obstructive lung disease, the distal airway resistance contributes to between 50 and 90% of the total lung resistance.[52] Van Brabandt and colleagues have concluded that the contribution of the distal airways to the total lung resistance has thus far been grossly underestimated and that the physiological outcome is largely dependent on the frequency used to measure the peripheral lung mechanics. They argued that this is because at high-frequency, low-amplitude oscillations the contribution of the distal airways to the total lung resistance is minimized, whereas at low-frequency, high-amplitude oscillations their contribution is maximized.[52]

More recently peripheral airways, including lung tissue, have been recognized as a predominant site of airflow obstruction in asthmatics.[5,8,53] In partitioning the central and peripheral airway resistance in awake humans, Yanai and colleagues have demonstrated a dramatically increased contribution of the distal airways to the total lung resistance in patients with moderate-to-severe asthma when compared to normals or to patients with mild asthma.[5] In these patients, lung function measurements were performed in a single lobe of the lung and it may be argued this is not an accurate depiction of total lung resistance; however, these values were similar to those measured throughout *postmortem* human lung specimens.[54] Furthermore, Wagner and colleagues have shown that in mild asthmatics with normal spirometry, the distal airway resistance was increased up to sevenfold when compared to controls and these measurements correlated with responsiveness to MCh.[55] In addition, asymptomatic asthmatics can exhibit significant increases in peripheral airway resistance, which is likely to be a result of distal lung inflammation. Computational analyses based on quantitative histology have shown the peripheral airways to account for the majority of airway hyperresponsiveness (AHR) amongst asthmatics.[7,56]

## The Distal Airways and the Lung Parenchyma as a Therapeutic Target in Asthma

The physiological and pathological evidences presented in this review unquestionably suggest that the small airways and the lung parenchyma are implicated in the pathogenesis of asthma. However, what is unclear at the moment is whether inhaled corticosteroids, the mainstay of asthma treatment, effectively treat this compartment of the lung or not. Although inhaled corticosteroids reduce airway inflammation in mild-to-moderate asthmatics,[57,58] they are ineffective in normalizing AHR or abolishing airway inflammation in severe asthmatics despite prolonged courses of treatment.[59,60]

Deposition studies have demonstrated that the majority of currently used inhaled corticosteroids are primarily deposited mostly in the central airways and not in the lung periphery.[61] Metered-dose inhalers, pressurized inhalers or dry-powder inhalers are not very efficient at depositing medication in the more peripheral airways of the lung. These delivery systems typically deliver no more than 30% of the inhaled dose to the distal sites of the lungs.[62] The challenge for the pharmaceutical companies is to improve the technology of aerosol delivery systems so as to ultimately allow delivery of anti-inflammatory drugs to the peripheral as well as the central inflammatory sites with minimal oropharyngeal deposition, thus enabling inflammation to be treated uniformly throughout the airways.

Better lung deposition of the steroids may be achieved by modification of propellants to produce finer and slower moving aerosols. The latest development is the introduction of CFC-free HFA (hydrofluoroalkane) propellants, which exhibit improved airway targeting when compared to the chlorinated fluorocarbon CFC (Chlorofluorocarbon) propellants.[63–65] HFA formulation delivers extra-fine particles (~1 mm diameter), which are deposited in the central as well as in the peripheral airways of the lung where they have equivalent control of the disease and improvement in lung function.[65,66]

The primary concern with HFA formulations however is the safety of the inhaled steroids and their potential systemic adverse effects in asthmatic patients. These safety concerns have been addressed in a number of studies.[67] Addition of LABA to inhaled corticosteroids has also improved the delivery of steroids particles to small airways and lung parenchyma, and this might explain to an extent the improved effectiveness of the combination treatment in moderate-to-severe asthma.

## Conclusion

There is accumulating evidence to suggest that airway inflammation occurs throughout the airway. Although the clinical significance of the small airways and the lung parenchyma in asthma is not yet known, it is possible that poorly controlled inflammation in the distal airways, which is not penetrated by conventional inhaled steroids, may contribute to accelerated decline in lung function and airway remodeling. Although the nature of airway wall remodeling has been reasonably well described in the large airways, relatively little however is known about structural remodeling of the distal airways.

There is a need to assess distal lung and parenchymal inflammation in all degrees of asthma. Accurate detection and early diagnosis of small airway dysfunction is important as treatment during early stages of the disease may be able to effectively reverse airway remodeling and progression to airway fibrosis and irreversible airway damage in mild-to-moderate asthmatics. There is also a need to identify an accurate way to assess distal airway inflammation noninvasively. The introduction of high-resolution computed tomography allows assessment of morphological changes in the distal airways that are associated with dysfunction too subtle to be identified on lung function testing alone.[66] This novel noninvasive imaging technique may prove to be important not only in the diagnosis of distal airway inflammation but also in helping us towards a better understanding of the role of the small airways and the parenchyma in pathogenesis of allergic asthma.

We have learned a great deal about the peripheral airways with the help of new physiological and biochemical technologies; however, there are many questions which still remain unanswered in this important area of research.
